# Case of Epilepsy Only during Pregnancy

**Published:** 1877-09-20

**Authors:** 


CASE OF EPILEPSY ONLY DURING PREGNANCY.—Dr. Gatewood has attended a negro woman in twelve confinements, who is afflicted with epileptic fits during each pregnancy. They begin when she is about a month advanced, and generally last about ten days at a time, and then cease for about twenty days, but only to recur and last ten days longer, and so on during the whole period of gestation.

